# National levels of human development and number of mental hospital beds

**DOI:** 10.1017/S2045796020000761

**Published:** 2020-09-08

**Authors:** Justin Metcalfe, Robert Drake

**Affiliations:** Westat, Behavioral Health and Health Policy, Behavioral Health and Health Policy, Lebanon, New Hampshire, USA

**Keywords:** Economic issues, inpatient psychiatry, mental health, psychiatric services, social and political issues

## Abstract

**Aims:**

The number of mental hospital beds per population varies widely across countries, and the reasons for this variation are not fully understood. Given that differences in disease prevalence do not explain variation in inpatient mental health care availability, we examined the relationship between mental hospital beds and national income, education and longevity as measured by the Human Development Index (HDI).

**Methods:**

We used an international dataset of social, economic and structural measures to conduct a mixed-effects longitudinal regression of predictors of the number of mental hospital beds per 100 000 in the overall population for 86 countries for years 2005–2015.

**Results:**

Our initial dataset contained 1881 observations consisting of 11 years of potential measurements across 171 countries. After eliminations based on missing data and subsequent imputation, the dataset for the final regression model included 946 observations over 86 countries. The primary predictors of a country's number of mental hospital beds were year, HDI and GINI coefficient, the latter being a measure of income disparity. Holding all other factors constant, the number of beds decreased 8% per year, reflecting the ongoing international trend of deinstitutionalisation. As hypothesised, higher HDI predicted more mental hospital beds. Every 0.1 increase in HDI (0–1.0) was associated with a 126% increase in the number of hospital beds at the sample's mean GINI index score of 38 (0–100). However, a strong interaction between HDI and the GINI coefficient indicated that a high level of income disparity attenuated the positive association between HDI and mental hospital beds. At a GINI index score of 48, every 0.1 increase in HDI was associated with a 71% increase in the number of hospital beds.

**Conclusions:**

As countries reduce the number of hospital beds over time, higher levels of economic disparity are associated with a reduction in the strength of the association between national prosperity and investment in mental hospitals. As power becomes increasingly concentrated, perhaps those with the least are more easily forgotten.

## Introduction

Inpatient hospitalisation is an important component of any functioning system of mental health care (Thornicroft and Tansella, 2013). As institutionalisation has fallen out of favour since the mid-20th century, the number of mental hospital beds has decreased in almost all countries (Allison *et al*., 2018), but the need for intensive care dedicated to those in acute distress clearly persists. Most countries continue to allocate a large proportion of their mental health budget to mental hospitals, despite potential alternatives such as crisis teams to provide intensive care (Drake and Wallach, 2019), so that determining the number of hospital beds per capita is one of the most fundamental policy decisions in any mental health system. Yet an evidence-based policy model does not exist. Instead, mental hospital bed numbers vary tremendously from country to country (Organisation for Economic Cooperation and Development, 2009), and policy decisions remain highly controversial (Drake and Wallach, 2019; O'Reilly *et al*., 2019).

All else being equal, determining inpatient care availability per population should be a function of both disease prevalence, which varies somewhat across countries (Richie and Roser, 2018), and the effectiveness of countries' community-based mental health systems (Drake and Wallach, 2019). However, all else is not equal. This simplistic zero-sum relationship, in which hospital-based care is traded for community-based care options, follows from the longstanding goal of mental health advocates and policy experts to reduce the reliance on mental hospitals by transferring resources from hospital- to community-based treatment, but it neglects the role of previously unmet need in this ongoing rebalancing. As unmet need varies with service availability and attitudes towards mental illness, it follows that cultural, social, economic and political factors may influence policy decisions that determine the allocation of hospital-based mental hospital beds. Understanding these non-clinical factors is critical if we are to advance towards a rational allocation of mental health expenditures.

We posited that resource availability is one critical predictor of the number of mental hospital beds. As a society's economic prosperity improves, self-investment in relatively resource-intense infrastructure such as education and health care, the latter including care devoted to mental health, should also increase (United Nations Development Program, 2019a). This resource availability is not simply a function of national income, but also of the ways in which available resources are, or are not, invested in the population. Our goal in this study was therefore to determine whether large-scale socio-economic factors drive the number of available mental hospital beds over the full range of national socio-economic conditions. We therefore examined the broad association between economic prosperity – measured primarily by the Human Development Index (HDI) – and the number of mental hospital beds per 100 000 population.

## Methods

### Overview

This exploratory study aimed to identify strong national socio-economic predictors of the number of mental hospital beds per 100 000 population. Potential predictors included public data describing a range of characteristics from 2005 to 2015.

### Countries

We started by collecting measures, where available, for 171 countries. After removing countries with large amounts of missing data, the final dataset came from 86 countries, including 26 countries that were members of the Organization for Economic Cooperation and Development for at least 1 year during the study period.

### Measures

*Outcome: mental hospital beds*. The average number of mental hospital beds per 100 000 population comes from periodic World Health Organization (WHO) Mental Health Atlas surveys. This count does not include beds in either general hospitals or community residential facilities (World Health Organization, 2004, 2005, 2011, 2014).

Potential confounders of the relationship between the number of mental hospital beds and the HDI included year, short-term economic health, prison population, ethno-linguistic fractionalisation, substance abuse and income disparity.

*Alcohol use disorder and drug use disorder disability-adjusted life years lost.* This is the number of healthy life-years lost to either premature death or time lived in poor states of health per 100 000. We used WHO estimates available by country for 2000, 2010 and 2015 (Mathers, 2018, World Health Organization Department of Information, 2018).

*Ethnic, language and religious fractionalisation.* These describe ethnic, linguistic and religious heterogeneity in a population. The measures represent the probability, on a scale of 0–1, that two individuals selected randomly from a population are members of different ethnic, linguistic or religious groups. Due to the known inverse correlation between ethno-linguistic fractionalisation and economic development, fractionalisation measures are commonly used as controls in analyses comparing national economic outcomes. Here we used an updated version of the measures (Alesina *et al*., 2003).

*GINI Coefficient*. The GINI Coefficient describes the degree to which income or, less often, consumption expenditure among individuals or households varies from a perfectly equal distribution. Based on household survey data from the World Bank and government statistical agencies, it is a continuous measure on a scale from 0 to 100, with 0 and 100 representing perfect equality and perfect inequality, respectively (World Bank Development Research Group, 2018).

*Human Development Index (HDI).* The HDI combines four components: mean years of school, expected years of school, gross national income per capita and life expectancy. It is a continuous measure with a bounded range of 0–1 (United Nations Development Program, 2018a, b; United Nations Development Program, 2019b).

*Mental health expenditures*. The proportion of total health care spending devoted to mental health care based on the 2005 and 2011 WHO Mental Health Atlas Surveys (World Health Organization, 2005, 2011).

*Population density*. Population per square kilometre of land area. Calculation is made based on midyear population and includes all residents (World Bank Group, 2019).

*Pretrial detention rate*. The proportion of people in prison who are being held prior to trial is based on the number of unsentenced detainees and the total number of prisoners (United Nations Office on Drugs and Crime, 2018a, b).

*Incarceration rate*. The number of people in prisons, penal institutions or correctional institutions for every 100 000 population comes from data reported to the United Nations by national statistical agencies. The counts do not include those held for administrative purposes (United Nations Office on Drugs and Crime, 2018a).

*Unemployment rate*. A measure of short-term economic health, this is the proportion of the labour force, defined as those either employed or unemployed and looking for work, who are unemployed (International Labor Organization, 2019).

### Statistical analyses

#### Data preparation

We gathered available measures for all countries. Many predictor values were unavailable or reported only intermittently from 2005 to 2015, yielding insufficient complete observations to conduct a regression analysis. Based on the assumptions that (1) national statistics represented populations rather than samples and were not subject to appreciable sampling error, and (2) these statistics represented underlying constructs subject to relatively smooth and gradual change over the relatively brief measurement period that would, in combination, enable the use of both extrapolated and interpolated values, we elected to impute missing predictor values, within individual countries, when at least one value for that predictor was available for the period of time in question (2005–2015). When a country did not have at least one value for each predictor over this period, we dropped that country. This criterion attenuated the sample from 171 to 86 countries. Next, we fit simple linear regression lines to every available country-specific predictor time series. We replaced missing values with the regression-predicted values. We log-transformed any predictors for which any extrapolated values fell out of range, specifically when values were close to zero, and regressed using the transformed values, translating the imputed values back into the original parameter space. We then inspected the trends modelled for each country's imputed measures to ensure that none of the extrapolated values used in the final model implied unreasonable or unrealistic deviations from the observed values upon which they were based. We scaled the predictors and outcome so that they occupied similar ranges, examined the distributions of each variable, and log-transformed highly skewed predictors to reduce the leverage of outliers. We also log-transformed the outcome variable (mental hospital beds per 100 000) to assure a normal distribution.

#### Model specification

To model the outcome, we chose a longitudinal mixed-effects model with a random intercept for every country. We used an identity link, normal distribution and Method = Laplace to create models based on the entire sample. We created our model in two stages. First, we included all potential predictors, log-transformed when highly skewed. Second, we created interactions of HDI with GINI, prison population, proportion of prison population in pretrial detention and mental health expenditures to examine whether the effects of these characteristics varied with HDI. We interpreted HDI as an informative proxy measure of OECD status. We evaluated the residuals for each model. Given the range of measurement quality and the variance-narrowing imputation technique we used, we considered significant any model parameters with extremely small *p*-values (<0.00001). We are making no causal – or, for that matter, final and definitive associative – inferences.

## Results

Our initial dataset contained 1881 observations consisting of 11 years of potential measurements for 13 variables, including the outcome, across 171 countries. After eliminations based on missing data and subsequent imputation, the dataset for the final regression model included 946 observations over 86 countries (Table 1). In the final model, we log-transformed prison population per 100 000, population density (population per square km) and the disability-adjusted life years associated with alcohol and drug use.

**Table 1.** Countries included in the analysis

Not OECD		OECD
Albania	Lesotho	Australia
Algeria	Lithuania	Belgium
Armenia	Madagascar	Canada
Azerbaijan	Malawi	Chile^a^
Bangladesh	Malaysia	Czechia
Bolivia	Mali	Estonia^a^
Botswana	Mauritania	Finland
Brazil	Mauritius	France
Bulgaria	Moldova	Germany
Burundi	Mongolia	Greece
Cameroon	Mozambique	Hungary
Chile^a^	Myanmar	Ireland
Colombia	Nicaragua	Israel^a^
Costa Rica	Pakistan	Italy
Cyprus	Papua New Guinea	Japan
Dominican Republic	Paraguay	Luxembourg
Ecuador	Peru	Mexico
Egypt	Philippines	Netherlands
Estonia^a^	Romania	Norway
Fiji	Senegal	Poland
Georgia	Slovenia^a^	Portugal
Ghana	Solomon Islands	Republic of Korea
Guatemala	Sri Lanka	Slovakia
Honduras	Tajikistan	Slovenia^a^
India	Tanzania	Spain
Indonesia	Thailand	United States of America
Iran	Togo	
Israel^a^	Tonga	
Kazakhstan	Tunisia	
Kyrgyzstan	Uganda	
Latvia	Uruguay	
Lebanon	Zimbabwe	

a Country joined the OECD during the observation period (2005–2015).

**Fig 1.**
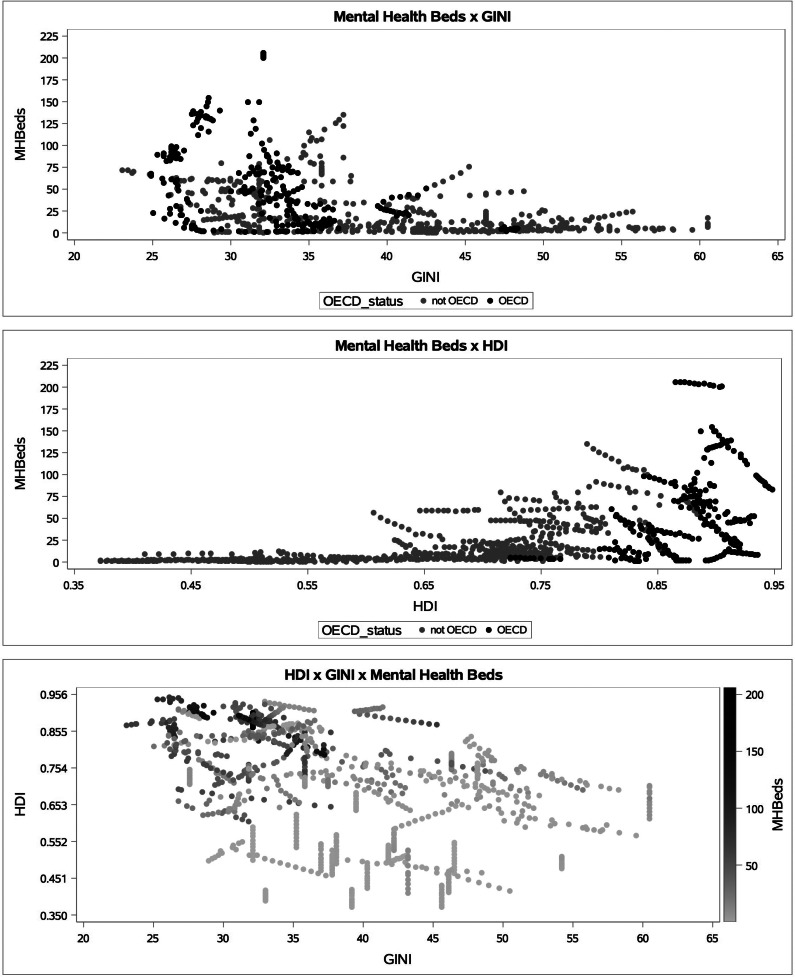
Scatterplots of mental health beds per 100 000 *v.* GINI and HDI from 2005 to 2015, stratified by OECD membership status, and HDI *v*. GINI *v*. mental health beds per 100 000.

Table 2 shows the characteristics of the included countries, both overall and stratified by OECD status. OECD status correlates highly with HDI (*ρ* = 0.68) and is a satisfactory proxy indicator of a country's gross national income per capita (*ρ* = 0.80). OECD nations have lower GINI (33 *v*. 40) and higher HDI (0.87 *v.* 0.65) than non-OECD nations. Figure 1 shows plots of GINI coefficient and Human Development Index versus mental health beds per 100 000 population and a plot of GINI coefficient versus Human Development Index Score.

**Table 2.** Characteristics of included countries, by OECD membership status

	All countries (*n* = 946)^a^		OECD countries (*n* = 266)^a^		Non-OECD countries (*n* = 680)^a^	
Predictor	Mean	STD	Mean	STD	Mean	STD
Alcohol use disorder DALY	304.34	241.36	343.20	219.63	289.14	247.85
Drug use disorder DALY	254.66	170.06	341.85	235.10	220.55	120.56
Ethnic fractionation	0.40	0.24	0.24	0.20	0.46	0.23
Expected years of school	13.34	2.87	16.36	1.58	12.16	2.35
GINI coefficient	37.99	8.17	32.85	5.53	39.99	8.17
Gross national income per capita ($)	16 387	15 163	35 850	12 660	8773	7140
Human development index	0.71	0.15	0.87	0.04	0.65	0.13
Language fractionation	0.37	0.27	0.23	0.20	0.42	0.27
Life expectancy	71.70	8.11	79.64	2.32	68.60	7.41
Mental health beds/100 000	28.36	37.55	54.50	50.67	18.13	24.25
Mental health expenditures (as per cent of total health exp)	3.79	3.08	6.21	3.14	2.77	2.41
Mean years school	8.72	3.02	11.45	1.44	7.66	2.79
Population density/sq. km	131.19	171.09	153.96	145.34	122.29	179.47
Prison population/100 000	152.50	105.98	154.28	132.59	151.80	93.65
Proportion of prisoners in pretrial detention	0.33	0.19	0.24	0.10	0.37	0.21
Religious fractionation	0.42	0.22	0.42	0.23	0.42	0.22
Unemployment	7.28	4.93	8.16	4.50	6.93	5.05

DALY, disability-adjusted life years lost.

a Sample size based on multiple annual measures for each country.

Table 3 shows the final model describing the relationships between the chosen predictors and the number of mental hospital beds per country. While the meaning of the *p*-value for each parameter in the context of this dataset is ambiguous, we treat it as a general indication of the strength of a predictor's association with the outcome. Thus, the parameters with the most significant associations with the number of mental hospital beds in a given country are the year, the HDI, the GINI coefficient and the interaction between the HDI and the GINI coefficient (*p* < 0.00001). Every year, the number of mental hospital beds decreases by 8% (exp(−0.082) = 0.92). The main effect of HDI assumes the values of all the variables with which it was interacted were zero, and the main effect of the GINI coefficient assumes an HDI of zero. We therefore zeroed each variable at its mean (Table 2) or, in the case of year, at 2010. For every 0.1 increase in HDI, the number of mental hospital beds increases 126% (exp(0.817) = 2.26), assuming a GINI coefficient of 38. However, at a GINI of 48, this is reduced to a 71% increase, assuming all other variables are held constant. For every 10-point increase in GINI coefficient, the predicted number of mental hospital beds decreases by 27% (exp(−0.316) = 0.73) at an HDI of 0.71, but at an HDI of 0.81, the number of mental hospital beds decreases by 45% (exp(−0.316 to 0.277) = 0.55).

**Table 3.** Models of mental health beds including all parameters

Main effects	Estimate	StdErr	*t* value	probt
Year^a^	−0.082	0.922	−14.884	0.00000
Prison population/100 000^a,b,c^	−0.108	0.898	−1.584	0.11354
Proportion in pretrial detention^c^	−0.040	0.961	−2.300	0.02171
Population/sq. km^a,b,c^	−0.188	0.828	−1.972	0.04892
Unemployment rate, %	−0.004	0.996	−0.694	0.48799
Human development index^a,c^	0.816	2.261	9.411	0.00000
Ethnic fractionalisation^c^	0.047	1.048	0.688	0.49135
Language fractionalisation^c^	0.029	1.030	0.491	0.62356
Religious fractionalisation^c^	0.077	1.080	1.513	0.13069
Alcohol use DALY^a,b,c^	−0.074	0.928	−0.444	0.65707
Drug use DALY^a,b,c^	−0.310	0.733	−1.517	0.12981
GINI coefficient^c^	−0.316	0.729	−4.294	0.00002
Mental health expenditures (% of total health care)^a^	0.029	1.029	2.209	0.02749
Interactions of HDI with				
Year^a^	−0.005	0.995	−1.721	0.08568
Prison population/100 000^a^^,^^b^^,^^c^	−0.086	0.917	−2.246	0.02496
GINI coefficient^c^	−0.277	0.758	−5.478	0.00000
Proportion in pretrial detention^c^	0.019	1.019	1.713	0.08708
Mental health expenditures^a^	0.013	1.013	1.653	0.09880

Some parameters log-transformed to compensate for skewed distributions (*N* = 946; df = 762).

a Variable zeroed (at 2010 for year and means for other variables – see Table 2).

b Variable log-transformed.

c Variable scaled (prison population, population density and DALYs/100; mental health beds/10; proportion pretrial, HDI and fractionalisation/0.1).

Based on both its components and correlates in the present dataset, the HDI represents a general description of social and individual health and social investment. It is positively correlated with mental health expenditures (*ρ* = 0.60) and disability-adjusted life-years lost per 100 000 due to drug use (*ρ* = 0.56), characteristics associated with wealthier countries. It is also positively correlated with prison population per 100 000 (*ρ* = 0.34), which itself lacks strong correlations with either wealth (*ρ* = 0.11 with gross national income per capita) or economic inequality (*ρ* = 0.18 with GINI coefficient). HDI is negatively correlated with ethnic fractionalisation (*ρ* = −0.48), language fractionalisation (*ρ* = −0.43), proportion of prison population in pretrial detention (*ρ* = −0.41) and GINI coefficient (*ρ* = −0.41).

The GINI coefficient is a measure of economic inequality. It correlates positively with proportion in pretrial detention (*ρ* = 0.38) and ethnic fractionalisation (*ρ* = 0.32), but negatively with mental health expenditures (*ρ* = −0.48) and, as previously mentioned, HDI (*ρ* = −0.41).

## Discussion

Our study found three primary predictors of the number of mental hospital beds per capita in a given country: time, HDI and GINI Coefficient. A downward year-to-year trend in the number of beds confirmed that the deinstitutionalisation movement, which began in the middle of the 20th century, remained ongoing up to 2015. Deinstitutionalisation included a shift in funding for mental hospital beds as countries developed community-based systems of care.

The number of mental hospital beds in a country also correlated strongly with both HDI and the GINI coefficient. Increasing levels of investment in a country's own population (HDI) was positively associated with an increasing number of available mental hospital beds, whereas higher economic inequality was negatively correlated with bed availability. Moreover, a strong interaction between the HDI and the GINI coefficient attenuated the positive association between HDI and the number of mental hospital beds in high-GINI countries.

The prominence of the HDI–GINI interaction in this analysis indicates a socio-economic dynamic that is more subtle than our primary hypothesis of a direct association between resource availability and mental hospital beds. In low-GINI nations, increased investment in education and physical health care (HDI) was associated with higher numbers of mental hospital beds, as hypothesised. Resources devoted to mental hospital beds appeared to be one aspect of and track with the general investment in population education and health care. By contrast, in countries with pervasive economic disparity (high GINI), we found no appreciable increase in the number of mental hospital beds in relation to a high HDI.

The parsimonious interpretation of this interaction is that extreme economic disparity reduces the extent to which resources are devoted to those residing at the social margins. Alternatively, it is possible that nations with both high GINI and HDI are allotting resources to community-based forms of care instead of mental hospital beds. Unfortunately, the data available to describe community-based care options are incomplete, and we elected not to use the measures available from the WHO Mental Health Atlas (mental health beds in general hospitals per 100 000 and community residential beds per 100 000), which provide incomplete descriptions of community-based services and would further limit the sample size and study period. However, previous research focusing on OECD nations has indicated that, in higher-HDI nations, community-based mental health care is positively correlated with mental hospital care (Perera, 2020). This correlation does not imply that increased investment in hospital-based care causes a similar increase in community-based options. Assuming that the observed correlation holds for nations with HDIs lower than those included in the aforementioned study, it seems more likely that lack of investment in mental health resources in countries with high GINI coefficients represents a natural consequence of wealth's unequal distribution.

As a step towards understanding national differences in the number of mental hospital beds, our analysis confirms the hypothesised strong relationship between a country's level of human development, which is highly correlated with national income, and the number of available mental hospital beds. Our analysis also indicates that this association is muted, but not eliminated, in countries with higher levels of income inequality. This result, in combination with the finding of a positive correlation between hospital- and community-based care, even in highly developed nations (Perera, 2020), suggests that neither resource availability nor clinical demand is the only driver of mental health services. This places the popular, zero-sum theory that a fixed number of mental health patients are distributed among community- and hospital-based care options in perspective. For this theory to be true, there must be a minimal unmet need for mental health care, however distributed. The strength of the association between socio-economic characteristics and the number of mental health beds implies that such conditions are not commonly met unless those socio-economic conditions are highly correlated with the need for mental health care. Only when national mental health systems are able to address the needs of greater proportions of their populations will the feedback between community- and hospital-based care options become a defining dynamic.

Yet, even in nations approaching the hypothesised zero-sum relationship between community and hospital care, other non-clinical factors beyond human development undoubtedly influence policy decisions regarding the balance of inpatient and outpatient resources, not least of which include large cultural differences in response to deviant behaviour and non-conformity. For example, some Asian countries, such as South Korea, with strong social stigma regarding mental illness have a bias towards inpatient care (Ng, 1997; Cho *et al*., 2009; Heo *et al*., 2019), while some European countries, such as Italy, have developed a strong system of shifting mental hospital beds to local general hospitals to counter stigma (Tansella, 1986; Barbui *et al*., 2018). Transitioning from national to local administration of hospitalisation may also impact the consistency of service quantity and quality (Barbui *et al*., 2018). Service areas also differ greatly in demographic characteristics. Some have large numbers of immigrants struggling with trauma and the stresses of a new language and culture (Alegría *et al*., 2017; Sangalang *et al*., 2019); major urban areas often stress residents with, for example, poor housing quality, pollution and lack of green space (Rautio *et al*., 2018); and rural areas may suffer from ongoing economic deterioration (Economic Innovation Group, 2016). It also is important to note that the conditions under which the zero-sum theory would be most apparent – specifically, a substantial period of time during which all of those in need of mental health care receive services in some form – are not known to have existed at any national level.

Developing an evidence-based model for planning the distribution of mental health resources is undoubtedly complex. Given the inherent limitations of population-level measures on causal inference, highly focused individual-level studies of relatively contained communities and their mental health systems may provide more robust insight into the question of how many mental hospital beds should be available to a community. Drawing samples from societies with a range of social characteristics would provide a means of studying the role of economic disparity and other non-clinical factors in mental health care.

### Limitations

The dataset used in this analysis of country-level measures came from a variety of sources. The methodologies used to generate each measure were of variable consistency, and certain useful measures (e.g. of community mental health resources) were unavailable for many countries. Furthermore, a large proportion of the dataset's observations had missing values, and we imputed these missing values using a simple imputation technique based on the assumption that all measures were population-level parameters subject to a minimal measurement error, as opposed to the more common assumption that measures are statistics describing the characteristics of samples drawn from populations. The absence of direct measures of community-based care is another important shortcoming, as it prevented any measure of alternative forms of care that may have offset the need for beds in mental hospitals. Given these limitations, our analysis only demonstrates broad associations, rather than causal relationships, between the measured parameters and the number of mental hospital beds in a country.

## Conclusions

The number of mental hospital beds in a given country represents an outdated and inefficient, if still common, expression of the capacity of a mental health system to care for those most in need of assistance. Ideally, mental hospital beds are available to cope with the actual need for such levels of care. This clinical need likely varies in accordance with multiple factors, including underlying rates of mental illness and the quality of community-based care options. Our analysis confirms that the number of mental hospital beds in a country correlates with other forms of social investment like education and health care. However, the concentration of income and, presumably, power in the hands of smaller portions of the population may reduce the likelihood that investment in mental hospital beds will track investments in education and overall health. In such circumstances, those with mental illness are among the least powerful and the first to be neglected.

## Data

All data used in this study are freely available to the public from the referenced sources.
